# Genetic diversity and spread dynamics of SARS-CoV-2 variants present in African populations

**DOI:** 10.1098/rsos.231326

**Published:** 2024-06-05

**Authors:** Desire Mtetwa, Tafadzwa Manjengwa, Zedias Chikwambi

**Affiliations:** ^1^ Department of Biotechnology, School of Health Science and Technology, Chinhoyi University of Technology, Private Bag 7724, Chinhoyi, Zimbabwe; ^2^ African Institute of Biomedical Science and Technology, 911 Chiedza Park, Boronia, Harare, Zimbabwe

**Keywords:** SARS-CoV-2, lineages, genomic diversity, COVID-19, spread dynamics

## Abstract

The dynamics of coronavirus disease-19 (COVID-19) have been extensively researched in many settings around the world, but little is known about these patterns in Africa. A total of 7540 complete nucleotide genomes from 51 African nations were obtained and analysed using the National Center for Biotechnology Information and Global Initiative on Sharing Influenza Data databases to examine the genetic diversity and spread dynamics of the severe acute respiratory syndrome coronavirus 2 (SARS-CoV-2) lineages circulating in Africa. Using various clade and lineage nomenclature schemes, we examined their diversity and used maximum parsimony inference methods to reconstruct the evolutionary hypotheses about the spread of the virus in Africa. According to this study, only 465 of the 2610 Pango lineages found to have existed in the world circulated in Africa three years after the COVID-19 pandemic, with five different lineages dominating at various points during the outbreak. We identified South Africa, Kenya and Nigeria as key sources of viral transmission among sub-Saharan African nations. These findings provide insights into the viral strains that circulate in Africa and their evolutionary patterns.

## 1. Introduction

In December 2019, severe acute respiratory syndrome coronavirus 2 (SARS-CoV-2) and the disease caused by the infection, named COVID-19, were originally identified in Wuhan, China, as a novel virus that produced a cluster of atypical pneumonia cases [1]. The outbreak soon became a global pandemic, resulting in a global public health emergency [2]. COVID-19 has affected all seven continents, with Africa being the least affected [3].

The first confirmed case in Africa occurred on 14 February 2020, in Egypt, while the first case reported in sub-Saharan Africa occurred on 27 February 2020, in Nigeria [4]. The first cases in other African countries, including Ghana, were recorded in March 2020 [5] on 12 March 2020. Globally, as of 21 February 2023, there have been 757.26 million confirmed cases of COVID-19, including 6.85 million deaths, reported to the World Health Organization (WHO) [6]. SARS-CoV-2 is the third novel coronavirus associated with significant outbreaks in the twenty-first century and the seventh coronavirus known to infect humans [7]. The first was SARS-CoV in 2003, which also appeared in China [8], and the second was the Middle East respiratory syndrome coronavirus (MERS-CoV) in 2012, which appeared in Saudi Arabia [8,9]. SARS-CoV-2 is more contagious but appears to have a lower case fatality rate (CFR) than SARS-CoV and MERS-CoV, both of which cause serious illnesses in humans [1,10].

The emergence of SARS-CoV-2 on the African continent necessitates a thorough examination of its genomic and evolutionary patterns. Comparative analysis of viral genome sequences is an extremely useful method for gaining insights into pathogen emergence and evolution. As a result, this study conducted an in-depth investigation into the epidemiology and evolution of SARS-CoV-2 in Africa to shed light on pandemic dynamics, investigate the number of variants circulating within Africa, and access the past deployment of control measures efficiency and inform future interventions on the African continent.

## 2. Methodology

### 2.1. Dataset mining and workflow

SARS-CoV-2 genome sequences collected from Africa were obtained from the National Center for Biotechnology Information (NCBI) [11] and Global Initiative on Sharing Influenza Data (GISAID) [12] databases on 26 February 2023. A total of 24 415 African sequences were retrieved from both databases to examine the number of lineages circulating within Africa. The two databases had only 8044 complete genome sequences combined from Africa, and these sequences, excluding those with low coverage using NextClade [13], were retrieved to determine spread dynamics. A total of 5908 sequences from 23 African countries are available in the NCBI database and 2137 sequences from 41 African countries are available in the GISAID database. The sequences were aligned using the online version of MAFFT [14] multiple sequence alignment tool [15] with Wuhan-Hu-1 (MN 908947.3) as the reference sequence, and sequences with more than 5.0% ambiguous letters were removed. Duplicates were removed using the goalign dedup software, and only high-quality African complete sequences remained (*n* = 7540).

### 2.2. Phylogenetic reconstruction

Using IQ-TREE multicore software version v1.6.12 [16] and NextClade [13], phylogenetic reconstruction of the dataset was performed. IQ-TREE was used in its default mode with Wuhan-Hu-1 (MN 908947.3) as the reference genome as an outgroup; the command ‘iqtree -s alignment_filename.fasta –m GTR+G’ was used. NextClade was also used in its default mode.

### 2.3. Lineage classification

PANGOLin, a Web application [17], was used to classify sequences into their lineages. The objective was to determine the most important SARS-CoV-2 lineages circulating in Africa from an epidemiological perspective, as well as the lineage dynamics within and across the African continent, as this naming system integrates genetic and geographic data on SARS-CoV-2 dynamics [17].

### 2.4. Phylogeographic reconstruction

Variants of Concern (VOC), Variants of Interest (VOI) and Variants Under Monitoring (VUM) were designated based on the WHO framework as of 20 January 2022. We included one lineage, Variant A, and labelled it the VOI for the purposes of this analysis. This lineage was included because it demonstrates the continued evolution of African lineages into potentially more transmissible variants. The VOI, VOC and VUM that emerged on the African continent were marked. These were Variant A (VOI), Beta and Omicron (VOC) and IHU Variant and Eta (VUM). Genome sequences of the five lineages were extracted from the NCBI database for phylogeographic reconstruction. An approach similar to that described above (including alignment using online MAFFT) was employed. Phylogeographic reconstruction for all variants circulating in Africa and all VOI, VOC and VUM were conducted using PASTML [18] and maps using QGIS. PASTML was used for coalescent analysis and/or ancestral state reconstruction.

## 3. Results

### 3.1. Genetic diversity and lineage dynamics in Africa

**Figure 1.**
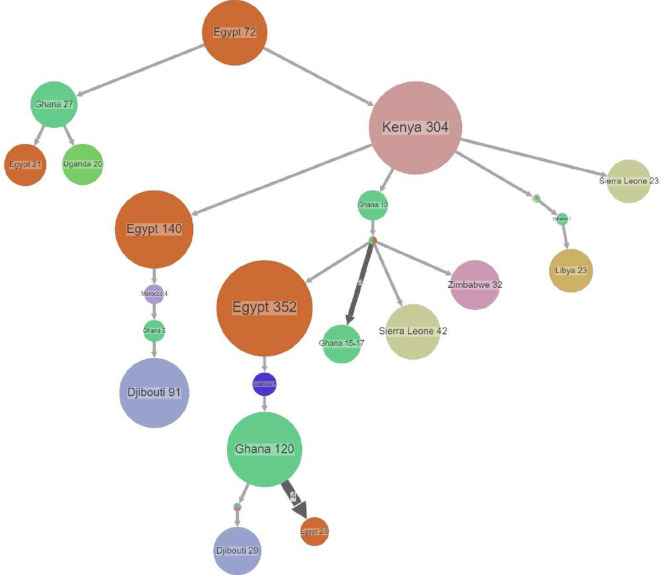
Spread dynamics reconstruction within Africa irrespective of the lineages. The figure shows compressed tree visualizations created by PastML using an F81-like model and MPPA. According to the map, various colours denote various countries. The sizes of the circles represent samples inside and the thickness of the arrow relates to a number of sequence IDs making up the circle. After entering Egypt, the virus spread to Kenya and Ghana. From there, there were imports and exports of the virus between African countries, and diversification events occurred across the continent.

The NextClade results showed that 465 of the 2610 Pango lineages circulated worldwide in Africa. Of the 8044 complete genomes retrieved from NCBI and GISAID, 7540 were retained after removing duplicates and sequences with more than 5% ambiguous letters from the dataset. We determined the import and export of viruses between African nations using ancestral location state reconstruction of dated phylogeny (figure 1). The early stage of the pandemic was marked by the dominance of lineage B.1, a large European lineage that caused most deaths in Italy (it approximately correlated with the northern Italian breakout in early 2020). Following the emergence of Beta in South Africa, it became the most common SARS-CoV-2 lineage in Africa during the second wave of the pandemic. Owing to the relaxation of travel restrictions, other variants of concern, such as Alpha, the UK lineage, and Delta, an Indian lineage, made their way into Africa and became dominant during the third wave of the pandemic. Eta and Gamma are some notable lineages that dominated during different waves of the pandemic. Omicron preceded the fourth pandemic wave and was first reported in South Africa and Botswana. Egypt reported the first case of SARS-CoV-2 infection in Africa (figure 1), which was of lineage B. This lineage was exported from other continents, primarily Europe, to other African countries.

**Figure 2.**
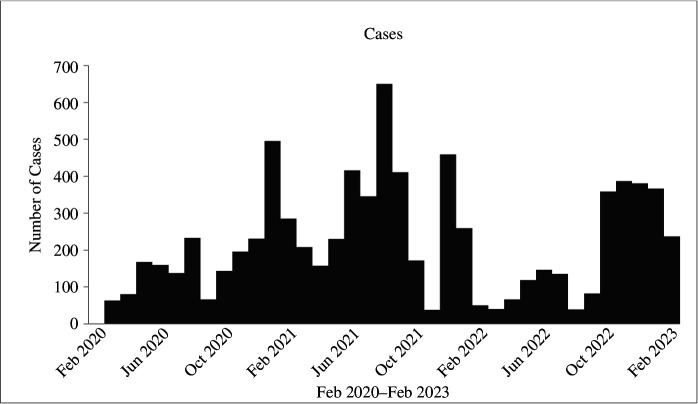
Visual presentation of epidemic waves and case rates through time in Africa.

There were distinct waves over time, according to a time series of case incidences in Africa (figure 2). The virus entered Africa in March 2020, marking the beginning of the first wave, with May 2020 seeing the highest number of cases. In July/August 2020, the Beta variant first appeared in South Africa, signalling the start of the second wave. August saw the greatest number of cases from this wave. The arrival of Delta, Alpha and Gamma variants marked the beginning of the third wave (figure 2). Owing to the easing of travel restrictions, the variants entered Africa in October 2020, November 2020 and January 2021, respectively. The third wave’s peak number of cases was noted in January 2021. Before the number of instances began to decline in February 2021, there was a noticeable surge from October 2020 to January 2021 (figure 2). In November 2021, Omicron emerged in South Africa marking the start of the fourth wave. The waves appeared to be defined by either newly introduced variants in populations from other populations or novel arising variants as a result of mutations.

**Figure 3.**
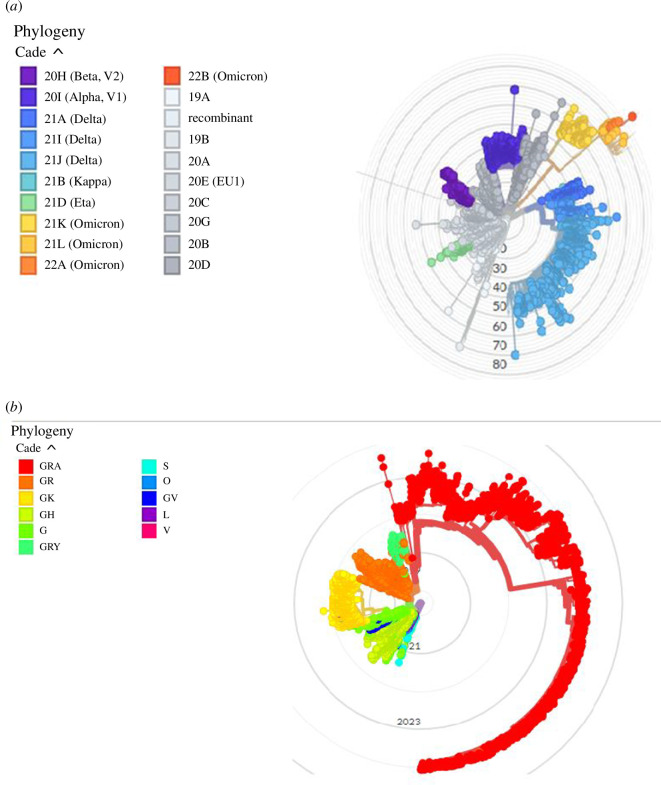
Phylogenetic tree reconstruction of clades circulating in Africa produced by NextClade (*a*) and GISAID (*b*). Different colours correspond to different clades as shown in the key (left).

NextClade detects mutations in other sequences from a reference sequence and uses these differences to classify sequences into clades or lineages. Figure 3*a* shows the genetic diversity of clades in the reference sequences. Different colours represent different clades that originate from different mutations, and the branches of the tree represent the distance between these clades from the original sequence. GISAID separates the clades of SARS-CoV-2 into S, O, L, V, G, GH, GR, GV and GRY (figure 3*b*). When the pandemic first started, the S and L clades circulated. At first, S remained common, whereas L divided into G and V. G went on to break into GR, GH, and eventually GV. After July 2020, GR separated into GRY. The mutations that led to their branching are represented by the letters [12].

Alternatively, Nextstrain divides the SARS-CoV-2 strains into 19A, 19B, 20A, 20B, 20C, 20D, 20E, 20F, 20G, 20H, 20I and 20J (figure 3*a*). The initial reference strain in these clades is 19B. 20J/501Y.V3 denotes the P.1 strain that originated and spread from Brazil; 20H/501Y.V2 denotes the B.1.351 strain that began in South Africa; and 20I/501Y.V1 is the B.1.1.7 variation that originated in Britain [13]. From figure 3, we can clearly elaborate that GISAID clade S (figure 3*b*) is the same as Nextclade 19B (figure 3*a*), while GISAID clades L, O and V correspond to Nextclade 19A. GISAID clades G, GH and GR correspond to Nextclade 20A, 20C and 20B, respectively.

### 3.2. Emergence and spread of new SARS-CoV-2 variants

We carried out a phylogeographic analysis of VOC Beta and Omicron, VUM Eta and IHU variant and one additional variant that arose and that we identified as VOI for this analysis as variant A to understand how some of the important SARS-CoV-2 mutations are spreading throughout Africa.

#### 3.2.1. Variants of concern

##### 3.2.1.1. Beta

**Figure 4.**
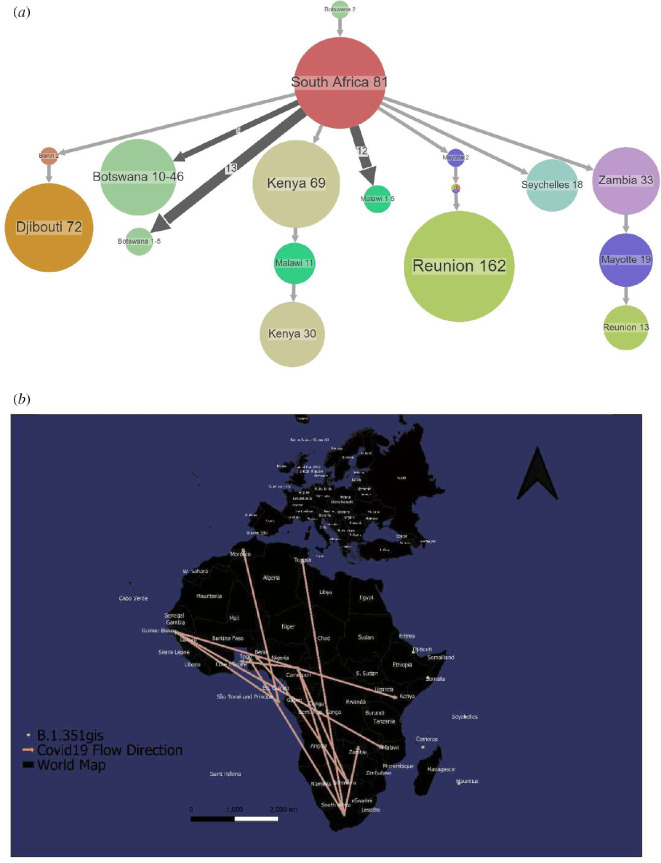
Spread dynamics reconstruction of Beta. (*a*) Compressed visualizations produced by PastML using MPPA with an F81-like model. The different countries are represented in the tree with different colours. The sizes of the circles represent samples inside and the thickness of the arrow relates to a number of sequence IDs making up the circle. (*b*) Spread dynamics of Beta on a geographical map.

Beta (B.1.351) was first sampled and reported in South Africa (figure 4*a*) in October 2020. When it first appeared in the Eastern Cape, the virus spread rapidly throughout South Africa, as the variant spread faster than previous variants of the virus (figure 4*b*). This variant was responsible for the second wave of the pandemic in South Africa. The variant had spread to bordering countries by November 2020 and to Djibouti, Malawi and Seychelles by December 2020. The predominant lineage in the majority of southern and eastern African populations by March 2021 was Beta. Beta also moved directly from southern Africa to eastern and central Africa (figure 4*a*).

##### 3.2.1.2. Omicron

**Figure 5.**
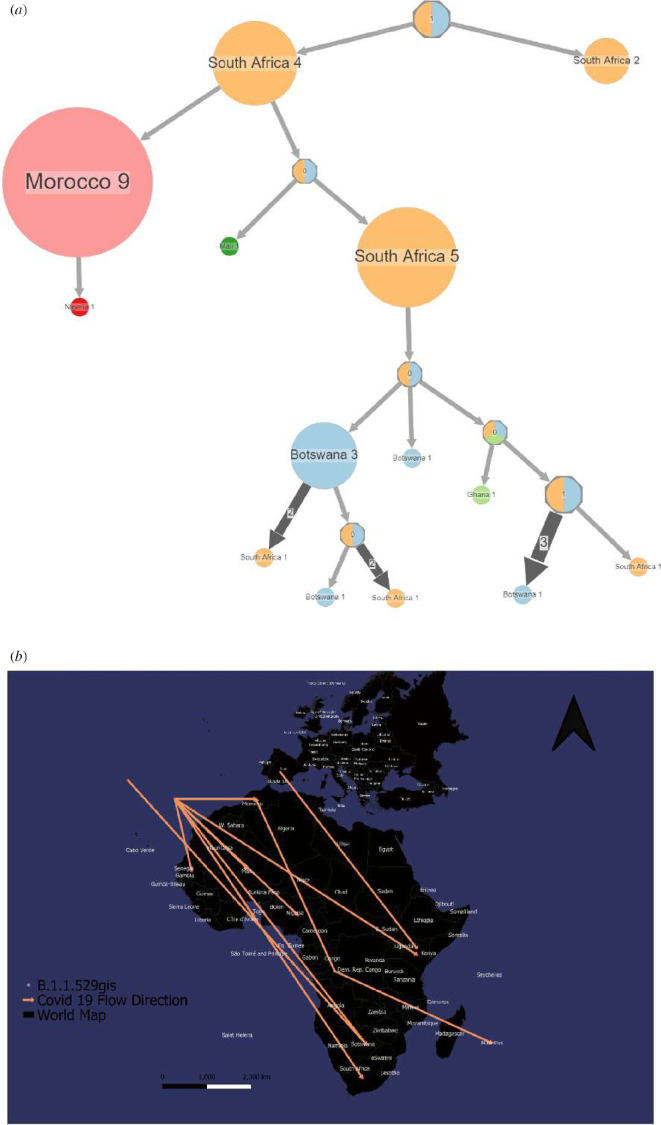
Spread dynamics reconstruction of Omicron. (*a*) Compressed visualizations produced by PastML using MPPA with an F81-like model. Different countries are represented in the tree with different colours. The sizes of the circles represent samples inside and the thickness of the arrow relates to a number of sequence IDs making up the circle. The Joint and MAP predictions are shown for the uncertain nodes. Although MAP and Joint disagree on the tree root, MPPA projections for all nodes are included in predictions (including those with unique MPPA prediction). (*b*) Spread dynamics of Omicron on a geographical map.

South Africa notified the WHO on 24 November 2021 that a new SARS-CoV-2 variant, Omicron, was discovered. Omicron was initially discovered in specimens collected in Botswana on 11 November 2021, and in South Africa on 14 November 2021. Since then, Omicron has been detected in samples collected in South Africa on 8 November 2021. VOC Omicron expanded into Botswana and South Africa’s neighbouring countries (figure 5*a*), then into northern and western Africa (Morocco and Nigeria), as well as into their neighbouring countries. Other transmissions from Botswana to Mali and Ghana have been reported. Obviously, in-between transmission and re-introduction of the variant to the southern countries occurred (figure 5*b*).

### 3.2.2. Variants under monitoring

#### 3.2.2.1. Eta

**Figure 6.**
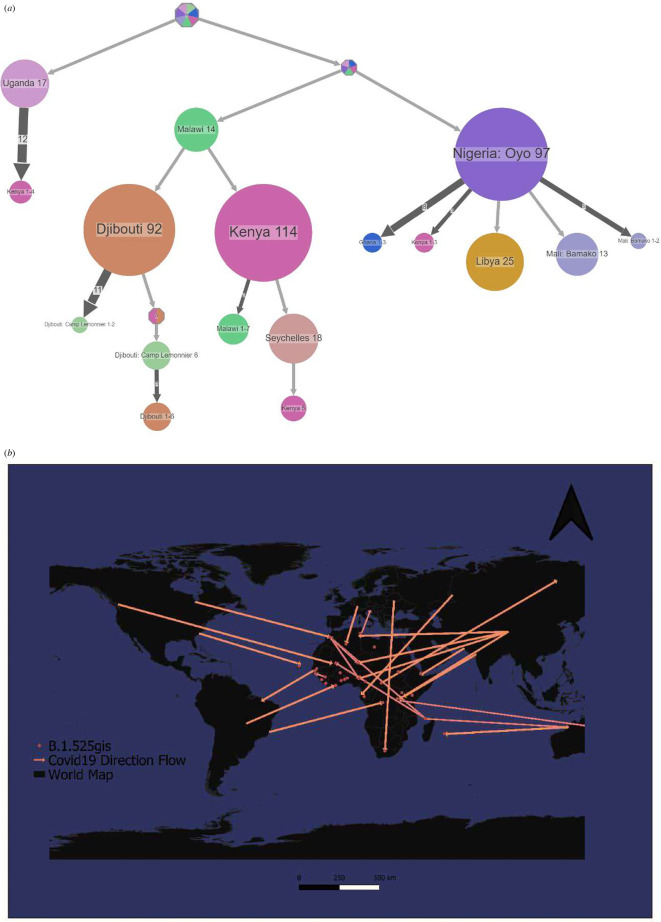
Spread dynamics reconstruction of Eta. (*a*) Compressed visualizations produced by PastML using MPPA with an F81-like model. Different countries are represented in the tree with different colours. The sizes of the circles represent samples inside and the thickness of the arrow relates to a number of sequence IDs making up the circle. The Joint and MAP predictions are shown for the uncertain nodes. MAP and Joint disagree on the tree root, but their predictions are included in MPPA predictions for all nodes (including those with unique MPPA prediction). (*b*) Spread dynamics of Eta on a geographical map.

The spread dynamics of Eta demonstrate the movement of Eta directly from Nigeria to neighbouring countries such as Ghana, Mali, Togo, Sierra Leone and East Africa such as Kenya and Djibouti, and northern Africa such as Libya (figure 6*a*). Eta was exported to other continents and was reintroduced back to Africa (figure 6*b*).

#### 3.2.2.2. IHU Variant

**Figure 7.**
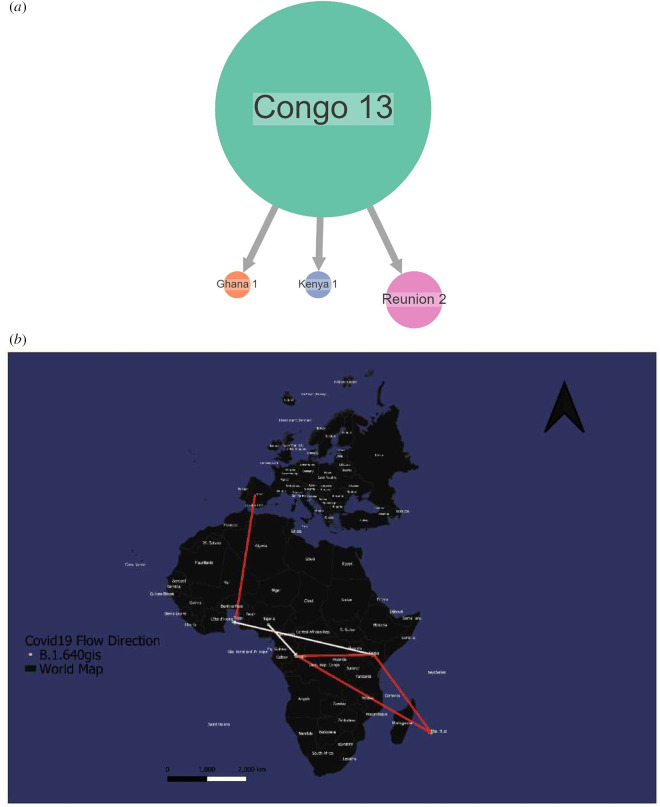
Spread dynamics reconstruction of IHU Variant. (*a*) Compressed visualizations created by PastML using a F81-like model and MPPA. Different countries are represented by different colours. (*b*) Spread dynamics of IHU Variant on a geographical map.

The IHU Variant originated in central Africa and was first reported in the Congo (figure 7*a*). It was directly transmitted to Ghana, Kenya and Reunion (figure 7*b*).

### 3.2.3. Variant of interest

#### 3.2.3.1. Variant A

**Figure 8.**
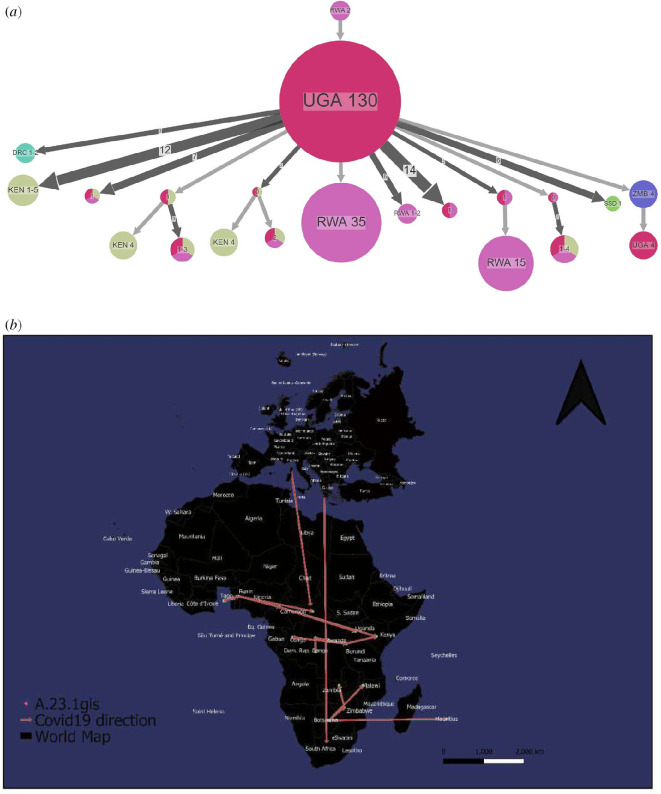
Spread dynamics reconstruction of Variant A. (*a*) Compressed visualizations produced by PastML using MPPA with an F81-like model. Different countries are represented in the tree with different colours. The sizes of the circles represent samples inside and the thickness of the arrow relates to a number of sequence IDs making up the circle. The Joint and MAP predictions are shown for the uncertain nodes. MAP and Joint disagree on the tree root, but their predictions are included in MPPA predictions for all nodes (including those with unique MPPA prediction). (*b*) Spread dynamics of Variant A on a geographical map.

For the sake of this investigation, we identified Variant A as a VOI because it offers excellent examples of the virus’s ongoing evolution in Africa. Since its discovery in September 2020, Variant A has regionally extended to neighbouring Rwanda and Kenya, as well as to the Democratic Republic of Congo (DRC), South Sudan and Zambia in the south (figure 8*a*). Figure 8*b* shows that Variant A was also exported outside Africa.

## 4. Discussion

The first SARS-CoV-2 case in Africa was reported in Egypt, followed by Nigeria, South Africa, Ghana and Kenya on the same day (12 March 2020). To date, Africa has contributed approximately 9.49 million cases and 175 000 deaths, which are 1.25 and 2.56% of the total cases and deaths reported worldwide, respectively [6]. A minimum of 84.88 million cases and 1.97 million deaths were recorded in 2020. In 2021, 205.88 million cases and 3.49 million deaths were reported while 442.53 million cases and 1.23 million deaths were reported in 2022 [6]. The highest numbers of cases were recorded in 2022, whereas 2021 recorded the highest number of deaths. This makes COVID-19 the number one infectious disease, surpassing tuberculosis. Although it seems that people have adapted to coexist with the virus and uplifting lockdown restrictions, these results show that SARS-CoV-2 is still circulating and killing.

Egypt, Kenya and South Africa appear to be key suppliers of imports to other African countries. Wilkinson *et al.* [19] found that this is likely owing to the fact that these three nations have the most deposited sequences and are among the first to report cases of SARS-CoV-2 in Africa. Lineage B was the first SARS-CoV-2 case reported in Egypt, indicating that its early introduction in Africa was an Asian lineage reported by China. The early stage of the pandemic was distinguished by the dominance of lineage B.1, a large European lineage (the origin of which roughly corresponds to the northern Italian outbreak early in 2020) rather than lineage B, which caused the establishment of the pandemic in China. According to Kamara and Essien [20], lineage B.1 was introduced multiple times to African countries in the early phase of the pandemic; thus, most introductions were predominantly from Europe rather than from Asia.

Our phylogeographic analysis of African countries revealed that after entering Egypt, the lineage developed into the B.1 lineage before extending to Kenya, Morocco and Ghana. Subsequently, the B.1 lineage was imported and exported across African countries, and the lineage diversified into numerous variants (diversification events) across the continent. Lineage A also entered the picture from other continents as air travel restrictions were relaxed, allowing for intra-country and occasional international viral movements between neighbouring countries, presumably via road and rail ties. Although some border posts between countries were closed during the initial lockdown period, others remained open to allow trade to continue. As a result, variants have evolved within and spread across continents.

When lineage Beta first appeared in South Africa, it quickly spread throughout the country because the variant was thought to propagate faster than previous virus versions. By March 2021, Beta had surpassed Variant B (B.1) as the dominant lineage in most southern and East African countries. During the second wave of the epidemic, it was the most frequently discovered SARS-CoV-2 lineage in Africa. This occurred because of the loosening of air and land travel limitations, which resulted in the arrival of various variants and lineages from other continents. VOC, such as Alpha of the UK lineage and Delta of the Indian lineage, made their way to Africa and became prominent during other pandemic waves. According to our phylogeographic reconstruction, Beta moved directly from southern Africa to eastern and central Africa. A discrete phylogeographic examination of a larger sample of viruses by Katella [21] implied that transmission to West Africa may have occurred via East Africa, with a probable European intermediary. BA.1, an alias of Omicron, was initially detected in South Africa and preceded the fourth pandemic wave. South Africa notified the WHO on 24 November 2021 that a new SARS-CoV-2 variant, Omicron, was discovered. Omicron was initially discovered in specimens collected in Botswana on 11 November 2021, and in South Africa on 14 November 2021.

Since then, South Africa has discovered Omicron in samples taken on 8 November 2021 [22]. The spike protein of the Omicron variant is characterized by at least 30 amino acid substitutions, three small deletions, and one small insertion. Owing to these mutations, there was a marked reduction in vaccine effectiveness, enhanced transmissibility and increased risk for reinfection and immune escape as compared with other variants like Delta. Consequently, Omicron was designated as a VOC. Omicron extended into Botswana and South Africa’s neighbouring countries, then into northern and western Africa (Morocco and Nigeria), as well as into their neighbouring countries (figure 5). Other transmissions from Botswana to Mali and Ghana have been reported. Clearly, the variety was reintroduced to southern and other countries during the time between transmissions. As a result, most southern African countries were placed on the blacklist and travel to other countries was prohibited.

Eta is a VOI defined by six substitutions in the spike protein and two deletions in the N-terminal domain. According to Katella [21], this variant was first sampled in the UK in mid-December 2020, but the phylogeographic reconstruction suggested that it originated in Nigeria in November 2020. Since then, it has spread throughout much of Nigeria and neighbouring Ghana. Our phylogeographic reconstruction also demonstrated the movement of Eta directly from Nigeria into neighbouring countries such as Ghana, Mali, Togo, Sierra Leone and eastern Africa such as Kenya and Djibouti and northern Africa, such as Libya (figure 6). The scope of this VOI distribution in the region is unknown because of poor sampling from neighbouring nations in western and Central Africa.

The IHU Variant is a variant that originated in central Africa. According to our phylogeographic reconstruction, it was first reported in Congo and directly transmitted to Ghana, Kenya and Reunion (figure 7). Given the small sample size of all African countries, the extent of the VUM spread in this region is unknown. What we know is that it has two sublineages, B.1.640.1 and B.1.640.2, and as of 17 February 2022, B.1.640.2 was a VOC because of its 46 mutations and 37 deletions in its genetic code, many affecting the spike protein. This sub-lineage was also dubbed as the IHU rose in France [23].

We designated Variant A as a VOI because it presents good examples of the continued evolution of the virus within Africa. According to Haseltine [24], the variant contains several mutations observed in variants of concern, as well as six unique substitutions. However, the variant does not have a similar origin to any of the variants of interest or concern, including Alpha, Beta, Gamma, Delta and Mu [24]. All variants shared a mutation that identified their common ancestry; Variant A, however, does not [25]. It has more in common with the A.30 variety, which was first discovered in Angola and may have originated in Tanzania. According to Haseltine [24], neither of these variants has a common ancestor with the other major strains, and the identification of two separate yet distantly related variations in East Africa is troubling in and of itself. The fact that these variations originate separately from all other variants worldwide, despite the fact that they lack the characteristic trio of mutations that link all other variants, illustrates the adaptability of SARS-CoV-2 to local conditions.

Lineage Variant A, characterized by three spike mutations (F157L, V367F and Q613H), was first detected in July 2020 in a Ugandan prison in Amuru [25]. The lineage was then transmitted to the Kitgum prison, possibly aided by the transfer of the prisoners. Subsequently, the A.23 lineage infiltrated the general population and spread to Kampala, introducing new spike mutations (R102I, L141F, E484K and P681R) as well as additional mutations in nsp3, nsp6, ORF8 and ORF9, prompting the creation of a new lineage classification, Variant A [25]. Since its discovery in September 2020, Variant A has spread regionally into neighbouring Rwanda and Kenya, as well as to the DRC, South Sudan and Zambia in the south (figure 8). However, according to Wilkinson *et al.* [19], the phylogeographic reconstruction of Variant A suggests that the introduction into Ghana may have occurred via Europe, whereas the introduction into southern Africa likely occurred directly from East Africa. This is consistent with epidemiological data indicating that the case discovered in South Africa was the result of contact with a person who had recently visited Kenya.

High rates of COVID-19 testing and consistent genomic surveillance in the south of the continent have resulted in early detection of VOCs, such as Beta and Omicron [26]. Since the discovery of these southern African variants, several other SARS-CoV-2 VOIs have emerged around the world, including on the African continent, including B.1.525 in West Africa and Variant A in East Africa. Strong evidence suggests that both VOIs are becoming more common in regions where they have been identified, implying that they may be more suitable than other variants in these regions. In the later months of 2020, the Beta variant moved from South Africa to neighbouring nations, reaching as far north as the DRC by February 2021, according to our phylogenetic analysis of the Beta branch.

This spread could have been aided by rail and road networks that connect South Africa’s ocean ports to economic and industrial centres in Botswana, Zimbabwe, Zambia and the DRC’s southern regions. The rapid and seemingly unhindered penetration of the virus into these countries implies that present land border regulations aimed at limiting the virus’s international transmission are ineffective and much needs to be done to implement and improve African land borders as far as epidemiology is concerned to contain such outbreaks in the future. From a public health standpoint, genetic surveillance is the only instrument in the pandemic preparedness toolset. However, genetic surveillance has not yet been effectively performed in Africa. The usefulness of molecular surveillance as a technique for monitoring pandemics relies heavily on ongoing and consistent sampling, rapid viral genome sequencing and timely reporting.

When this is accomplished, molecular surveillance can be used to detect the shifting pandemic traits early. Molecular monitoring data can inform public health interventions when such changes occur. In this context, molecular surveillance data collected by most African countries are less useful than they could be. For example, in some circumstances, the time lag between when virus samples are acquired and when sequences for these samples are stored in sequence repositories is so great that the primary utility of the genomic surveillance data is lost. Depending on the laboratory or country, numerous variables contribute to this lag: (i) lack of reagents owing to global supply chain disruptions, (ii) lack of equipment and infrastructure within the source country, (iii) lack of technical skills in laboratory methods or bioinformatics support, and (iv) reluctance of health officials to share data. To determine the genetic properties of currently circulating viruses in these nations, more recent sampling and fast reporting are required.

As a result, our study’s fundamental flaw is the patchiness of the African genetic monitoring data. It is worth noting that phylogeographic reconstruction of viral propagation is strongly reliant on sampling, with the caveat that the exact paths of viral migration between nations cannot be deduced if connecting countries are not sampled. Furthermore, uneven sampling between African countries is certainly biasing our efforts to recreate SARS-CoV-2 migration dynamics across the continent. It is no surprise that we identified South Africa, Kenya and Nigeria as key sources of viral transmission between sub-Saharan African nations because they had the most SARS-CoV-2 genomes sampled and sequenced. The strength of national public health programmes to embrace genomic surveillance as a tool for preventing the appearance and spread of dangerous variants determines their reliability as a strategy for preventing the emergence and spread of dangerous variants. Ishikawa *et al.* [18] stated that, as in most other parts of the world, the success of genomic surveillance in Africa requires more samples to be tested for COVID-19, with a higher proportion of positive samples being sequenced within days of sampling.

The degree of dissemination of specific variations in the region is unknown because of limited sampling across all African countries. Some variants were sequenced to exhaustion and were deposited in databases, whereas others, such as IHU Variant and Eta, had relatively few sequences deposited in databases. Some countries have a considerable number of sequences deposited in sequence repositories, whereas others have few or no sequences. As a result, the results of our study were skewed. PASTML, which we used to perform phylogeographic reconstruction, also required caution with sampling bias, and it was necessary to subsample sequences to better represent the number of declared cases by location and/or use of additional data, such as travel history, or phylogeographic predictions would be incorrect. Complete genome sequencing of SARS-CoV-2 is important for obtaining useful information about the viral lineages, variants of interest, and variants of concern; however, only approximately 32% of the nucleotide sequences deposited in the NCBI database from Africa are complete, with 68% partial nucleotide sequences.

As a continent, we need to be able to deposit complete genomic sequences because most researchers only take complete genomes for these types of studies. Perhaps because of this, we discovered that there are fewer lineages circulating throughout Africa than actually.

## 5. Conclusion

Africa has been the least-affected continent for several reasons. This may be owing to skewed statistics being reported or the fact that many people have turned to using ethnobotanical treatments to treat COVID-19 symptoms because Africa has a strong tradition of using plants. This might be because African populations have lower rates of comorbid conditions and are older than those of other continents. Our findings suggest that SARS-CoV-2 variants circulating in Africa follow unique patterns of evolution and transmission. We also found that all variants shared a mutation that identified common ancestry. However, Variant A does not, it haing more in common with the A.30 variety, which was first discovered in Angola, and may have originated in Tanzania. Neither of these variants had a common ancestor with other major strains. The fact that these variations originate separately from all other variants worldwide, despite the fact that they lack the characteristic trio of mutations that link all other variants, illustrates the adaptability of SARS-CoV-2 to local conditions. Since the discovery of the southern African variants Beta and Omicron, several other VOIs have emerged worldwide, including those on the African continent, specifically in West Africa (Eta) and East Africa (Variant A). According to the available data, both VOIs are clearly increasing in frequency in the areas where they have been detected, implying that they may be more suitable than other variants in these regions, thus suggesting that SARS-CoV-2 variants are specific to geographic conditions. Routine genomic surveillance of the variants, employing phylogenetic and phylodynamic analyses with wider coverage and higher resolution, is essential to provide insight into the effectiveness of different intervention strategies against the pandemic. The effectiveness of molecular surveillance as a method for tracking pandemics strongly depends on continuous and reliable sampling, rapid viral genome sequencing and prompt reporting, all of which must be improved in Africa. Additionally, the pandemic outbreak revealed that current land border regulations aimed at limiting the virus’s international transmission are ineffective, and much needs to be done to implement and improve African land borders as far as epidemiology is concerned to contain such outbreaks in the future. We also need universal or common criteria for sample selection, library preparation and sequencing platforms, bioinformatics workflows and data interpretation to ensure consistent data quality standards across public databases.

## Data Availability

The datasets generated and analysed during the current study are publicly available at [27]. Data is available in the supplementary material [28].
